# Contemporary data on low-density lipoprotein cholesterol target value attainment and distance to target in a cohort of 57,885 statin-treated patients by country and region across the world

**DOI:** 10.1016/j.dib.2016.09.037

**Published:** 2016-09-29

**Authors:** Anselm K. Gitt, Dominik Lautsch, Jean Ferrieres, John Kastelein, Heinz Drexel, Martin Horack, Philippe Brudi, Brecht Vanneste, Peter Bramlage, Francois Chazelle, Vasilisa Sazonov, Baishali Ambegaonkar

**Affiliations:** a Klinikum der Stadt Ludwigshafen, Medizinische Klinik B, Ludwigshafen, Germany; b Stiftung Institut für Herzinfarktforschung, Ludwigshafen, Germany; c Merck & Co., Inc., Kenilworth, NJ, USA; d Toulouse University School of Medicine, Toulouse, France; e Department of Vascular Medicine, Academic Medical Center/University of Amsterdam, Amsterdam, The Netherlands; f Department of Medicine and Cardiology, Academic Teaching Hospital Feldkirch, Feldkirch, Austria; g Vorarlberg Institute for Vascular Investigation and Treatment (VIVIT), Feldkirch, Austria; h Private University of the Principality of Liechtenstein, Triesen, Liechtenstein; i Drexel University College of Medicine, Philadelphia, PA, USA; j Institute for Pharmacology and Preventive Medicine, Mahlow, Germany

**Keywords:** Low-density lipoprotein cholesterol, Treatment target, Global, Region, Statins

## Abstract

Data presented here refer to 57,885 patients on lipid-lowering statin therapy from the Dyslipidaemia International Study (DYSIS) registry. Subjects were divided into 3 discrete subsets: those at very high-risk, high-risk, and non-high-risk for cardiovascular events, with assigned low density lipoprotein cholesterol (LDL-C) targets of 70 mg/dl, 100 mg/dl and 115 mg/dl, respectively. Overall, the highest proportion of patients meeting their LDL-C target was seen in the UAE and Kuwait (49.5%), while the lowest was seen in Germany (14.3%). The smallest median distance to target was documented in Canada (18.8 mg/dl), and the largest in the Baltics (42.1 mg/dl). Interpretation and discussion of this data can be found in the manuscript entitled “Low-density lipoprotein cholesterol in a global cohort of 57,885 statin-treated patients” (Gitt et al., 2016) [1].

**Specifications Table**

**Table:** 

Subject area	Biology
More specific subject area	Dyslipidemia and cardiovascular risk
Type of data	Tables
How data was acquired	Worldwide survey
Data format	Analyzed
Experimental factors	Observational registry
Experimental features	Comparison of LDL-C target achievement and distance to target in patients on chronic statin therapy at differing risk of cardiovascular events.
Data source location	Institut für Herzinfarktforschung, Ludwigshafen, Germany
Data accessibility	Data are included in this article

**Value of the data**

• These data are gathered from a large, worldwide registry and insights are therefore applicable to physicians globally.
• As data are stratified by individual country, target attainment within different national healthcare systems can be compared and potential improvements made based on the experience of more successful countries.
• Data can be used as a basis from which to launch further studies investigating optimal treatment regimes for patients at risk of cardiovascular events, both globally and within individual countries.

## 1 Data

Table 1 displays the proportion of patients attaining risk based target LDL-C values in a global cohort, overall and divided by country and region of the world. Table 2 displays the median distance to risk based treatment targets in a global cohort, overall and divided by country and region of the world.

## 2 Experimental design, materials and methods

The methodology for DYSIS (a cross-sectional, observational, multicenter registry) has been described elsewhere [1]. Briefly, statin-treated (mono/combination therapy) outpatients were consecutively enrolled at multiple centers located in 30 different countries worldwide. The study was approved by the relevant ethics committees and carried out in agreement with local laws.

Inclusion criteria were as follows: 1) provision of written informed consent, 2) availability of a fasting blood lipid profile taken within 6–12 months of study entry, at which time the participant had been on statin therapy for a minimum of 3 months 3) aged ≥45 years and on statin therapy at time of inclusion 4) not participating in a clinical trial.

The 2011 ESC/EAS guidelines on treatment of dyslipidemia were used for stratification of patients into risk categories [2]. Patients considered at “very high risk” of cardiovascular events were those diagnosed with CHD, diabetes, chronic kidney disease, and/or peripheral artery disease, whereas those with markedly elevated single risk factors such as total cholesterol >310 mg/dl or severe hypertension (SBP≥180 and/or DBP≥110 mmHg) were determined to be “high-risk”. All other patients were determined to be “not high-risk”. LDL-C targets of 70 mg/dl, 100 mg/dl, and 115 mg/dl were assigned to very high-, high- and non-high-risk patients, respectively, in accordance with 2011 ESC/EAS guidelines.

Serum lipid levels were obtained from the most recent blood test for each patient, and data on lipid-lowering agents (statin type, dose, and other concomitant lipid-modifying therapies) that were being taken by the patient at that time were documented. Simvastatin was used as a reference to calculate the relative potency of other statins for comparison [3]. A central web-based database at the Institut für Herzinfarktforschung, Ludwigshafen, Germany was used to collect and store the data.

The SAS^©^ statistical package, Version 9.3 (SAS Institute, Cary, North Carolina, USA) was used for data analysis purposes. Data were processed and presented as percentages (*n*/*N*) or medians (IQR), all of which were based one the number of patients with data for a particular case available. Categorical variables were compared by Chi-squared tests and continuous variables by Mann–Whitney–Wilcoxon (two-tailed) or Kruskal–Wallis (three-tailed) tests. In terms of distance from target, a smaller value was considered to represent greater target achievement, while a larger value represented poorer target achievement. *P*-values ≤0.05 were considered significant.

## Figures and Tables

**Table 1:** Proportions of patients attaining their target LDL-C values.

Country (*n*)	Total	Very high-risk^a^	High-risk^a^	Non-high-risk^a^	*P*-value
	% (*n*/*N*)	% (*n*/*N*)	% (*n*/*N*)	% (*n*/*N*)	
**Europe/Canada/Israel**					
Austria (*n*=881)	15.9 (123/772)	12.9 (85/657)	20.7 (6/29)	37.2 (32/86)	<0.0001
Baltics (*n*=1797)	15.9 (282/1779)	10.9 (151/1386)	20.5 (24/117)	38.8 (107/276)	<0.0001
Belgium (*n*=909)	35.7 (310/868)	21.6 (116/536)	40.5 (34/84)	64.5 (160/248)	<0.0001
Canada (*n*=2436)	45.6 (1098/2410)	40.7 (787/1933)	50.3 (79/157)	72.5 (232/320)	<0.0001
Denmark (*n*=933)	37.7 (338/897)	30.2 (196/650)	45.4 (49/108)	66.9 (93/139)	<0.0001
France (*n*=4192)	20.6 (835/4061)	14.4 (385/2677)	16.6 (50/302)	37.0 (400/1082)	<0.0001
Germany (*n*=4216)	14.3 (555/3879)	11.2 (371/3300)	18.9 (44/233)	40.5 (140/346)	<0.0001
Greece (*n*=755)	17.8 (132/741)	9.2 (42/456)	19.1 (9/47)	34.0 (81/238)	<0.0001
Ireland (*n*=900)	43.5 (376/865)	35.9 (222/618)	58.6 (51/87)	64.4 (103/160)	<0.0001
Israel (*n*=100)	29.1 (223/766)	20.3 (121/597)	49.0 (24/49)	65.0 (78/120)	<0.0001
Italy (*n*=766)	30.7 (206/671)	22.7 (95/419)	29.4 (15/51)	47.8 (96/201)	<0.0001
The Netherlands (*n*=1199)	30.8 (354/1151)	27.4 (279/1019)	34.1 (15/44)	68.2 (60/88)	<0.0001
Norway (*n*=957)	29.2 (247/847)	18.5 (112/607)	46.2 (48/104)	64.0 (87/136)	<0.0001
Portugal (*n*=910)	20.4 (144/706)	11.1 (46/415)	25.0 (13/52)	35.6 (85/239)	<0.0001
Russia (*n*=1585)	17.0 (189/1114)	12.2 (118/967)	30.3 (10/33)	53.5 (61/114)	<0.0001
Slovakia (*n*=926)	24.5 (226/923)	16.5 (121/733)	37.3 (19/51)	61.9 (86/139)	<0.0001
Slovenia (*n*=766)	23.6 (178/755)	19.1 (122/640)	25.6 (10/39)	60.5 (46/76)	<0.0001
Spain (*n*=3664)	16.7 (580/3463)	10.1 (221/2186)	17.9 (48/268)	30.8 (311/1009)	<0.0001
Sweden (*n*=958)	27.6 (223/807)	21.4 (141/660)	45.3 (34/75)	66.7 (48/72)	<0.0001
UK (*n*=1315)	40.9 (426/1041)	38.3 (353/922)	50.0 (13/26)	64.5 (60/93)	<0.0001

**Middle East/Africa**					
Egypt (*n*=1457)	18.8 (260/1384)	13.2 (157/1188)	28.2 (11/39)	58.6 (92/157)	<0.0001
Lebanon/Jordan (*n*=603)	41.6 (221/531)	33.0 (120/364)	37.1 (13/35)	66.7 (88/132)	<0.0001
UAE and Kuwait (*n*=299)	49.5 (135/273)	44.9 (105/234)	40.0 (2/5)	82.4 (28/34)	<0.001
Saudi Arabia (*n*=1263)	32.5 (388/1194)	26.4 (276/1045)	52.9 (9/17)	78.0 (103/132)	<0.0001
South Africa (*n*=1029)	48.6 (478/984)	39.9 (288/722)	66.7 (60/90)	75.6 (130/172)	<0.0001

**Asia**					
China (*n*=22,369)	31.4 (7006/22,345)	23.9 (4067/17,022)	46.4 (687/1481)	58.6 (2252/3842)	<0.0001
**Total** (*n*=57,885)	28.1 (15,533/55,227)	21.7 (9097/41,953)	38.0 (1377/3623)	52.4 (5059/9651)	<0.0001

a Corresponding LDL-C targets for very high-, high- and non-high-risk patients were <70 mg/dl, <100 mg/dl and 115 mg/dl, respectively.

**Table 2:** Median distance to treatment targets.

Country (*n*)	Total^a^ mg/dl (IQR)	Very high-risk^b^ mg/dl (IQR)	High-risk^b^ mg/dl (IQR)	Non-high-risk^b^ mg/dl (IQR)	*P*-value
**Europe/Canada/Israel**					
Austria (*n*=881)	33.0 (18.0, 58.0)	33.0 (19.0, 58.0)	31.0 (9.0, 67.0)	25.5 (9.0, 56.0)	0.06
Baltics (*n*=1797)	42.1 (20.5, 71.9)	42.5 (21.6, 73.9)	33.0 (12.9, 69.4)	38.1 (15.3, 60.9)	<0.05
Belgium (*n=*909)	24.3 (11.0, 44.0)	27.5 (14.0, 47.0)	14.5 (7.0, 30.0)	14.0 (5.0, 33.5)	<0.0001
Canada (*n*=2436)	18.8 (8.5, 34.4)	18.9 (8.5, 34.4)	17.7 (6.3, 33.4)	17.1 (8.4, 31.9)	0.79
Denmark (*n*=933)	24.2 (11.2, 42.1)	26.7 (13.9, 42.1)	22.6 (8.3, 46.9)	16.5 (4.9, 28.9)	<0.01
France (*n*=4192)	35.0 (18.0, 58.0)	38.0 (19.0, 61.0)	36.5 (17.0, 61.0)	28.0 (15.0, 48.0)	<0.0001
Germany (*n*=4216)	38.0 (20.0, 61.0)	39.0 (22.0, 62.0)	31.0 (14.8, 54.0)	28.1 (15.0, 45.9)	<0.0001
Greece (*n*=755)	37.0 (20.0, 60.0)	40.0 (22.0, 65.0)	35.0 (21.0, 50.0)	28.0 (15.0, 50.0)	<0.01
Ireland (*n*=900)	25.5 (11.2, 46.0)	26.7 (12.6, 46.0)	26.8 (14.7, 44.4)	19.6 (8.7, 38.1)	0.13
Israel (*n*=100)	21.0 (9.0, 38.0)	21.3 (9.0, 39.0)	23.0 (12.0, 29.0)	13.0 (7.0, 29.0)	0.13
Italy (*n*=766)	29.0 (13.0, 53.0)	32.0 (17.0, 55.5)	22.5 (7.0, 61.5)	22.0 (9.0, 37.0)	<0.001
The Netherlands (*n*=1199)	26.7 (11.6, 42.5)	26.7 (11.2, 42.1)	27.6 (13.7, 46.9)	31.8 (10.1, 39.7)	0.83
Norway (*n*=957)	30.5 (15.1, 49.9)	30.5 (18.9, 51.0)	16.0 (4.4, 31.7)	16.5 (4.9, 47.4)	<0.0001
Portugal (*n*=910)	39.5 (21.0, 60.0)	42.0 (23.0, 65.0)	35.0 (23.0, 56.0)	33.0 (16.0, 54.0)	<0.01
Russia (*n*=1585)	38.3 (18.9, 63.0)	38.3 (18.9, 65.0)	26.8 (8.3, 66.7)	28.5 (10.3, 42.4)	<0.01
Slovakia (*n*=926)	37.1 (18.2, 65.3)	38.7 (19.3, 68.4)	35.3 (16.4, 66.7)	18.8 (8.0, 31.9)	<0.0001
Slovenia (*n*=766)	38.3 (18.9, 69.2)	39.2 (18.9, 69.2)	23.7 (12.1, 54.7)	30.8 (16.5, 47.4)	0.05
Spain (*n*=3664)	39.0 (20.0, 64.0)	42.0 (23.0, 69.0)	39.5 (19.5, 61.0)	32.0 (14.0, 54.0)	<0.0001
Sweden (*n*=958)	26.7 (15.1, 46.0)	26.7 (15.1, 46.0)	27.6 (12.1, 46.9)	12.6 (8.7, 22.3)	<0.01
UK (*n*=1315)	19.3 (11.2, 36.3)	19.3 (11.2, 35.6)	28.4 (4.4, 40.8)	24.2 (4.9, 51.3)	0.98

**Middle East/Africa**					
Egypt (*n*=1457)	40.0 (20.0, 71.0)	42.0 (20.0, 75.0)	38.5 (22.0, 65.0)	29.0 (13.0, 47.0)	<0.01
Lebanon/Jordan (*n*=603)	30.0 (13.0, 58.0)	30.0 (13.0, 59.5)	25.5 (11.0, 37.0)	27.0 (13.5, 57.5)	0.37
UAE and Kuwait (*n*=299)	25.0 (9.0, 41.0)	25.0 (9.0, 42.0)	23.0 (2.0, 88.0)	11.5 (5.0, 24.0)	0.42
Saudi Arabia (*n*=1263)	34.0 (15.1, 56.0)	37.0 (17.0, 57.6)	13.5 (7.1, 24.0)	11.0 (6.0, 22.0)	<0.0001
South Africa (*n*=1029)	30.3 (14.3, 52.6)	30.5 (14.3, 53.7)	25.1 (13.7, 43.1)	34.7 (13.0, 53.2)	0.54

**Asia**					
China (*n*=22,369)	33.1 (15.5, 56.8)	34.8 (16.6, 59.5)	24.9 (11.8, 45.8)	24.2 (10.7, 43.5)	<0.0001
Total (*n*=57,885)	33.0 (15.8, 57.0)	34.4 (17.0, 59.0)	28.0 (12.1, 51.2)	25.8 (12.0, 47.0)	<0.0001

a Total refers to the distance from individual treatment targets, irrespective of group.

b Corresponding LDL-C targets for very high-, high- and non-high-risk patients were <70 mg/dl, <100 mg/dl and 115 mg/dl, respectively (given distance from target is relative to these values).
