# Supplementary material for: Contemporary data on low-density lipoprotein cholesterol target value attainment and distance to target in a cohort of 57,885 statin-treated patients by country and region across the world
Source: Data Brief. 2016 Sep 29;9:616–20. doi: 10.1016/j.dib.2016.09.037 (PMC5066206; doi:10.1016/j.dib.2016.09.037)
Supplement: Supplementary file 1 — Supplementary material [file mmc1.pdf]

AUTHOR DECLARATION TEMPLATE

Gitt AK et al.: "Contemporary data on low-density lipoprotein cholesterol target value attainment and distance to target in a cohort of 57,885 statin-treated patients by country and region across the world" *Data in Brief* 2016

We wish to draw the attention of the Editor to the following facts which may be considered as potential conflicts of interest and to significant financial contributions to this work:

Dr. Gitt reports to have received consultancy fees and lecture fees by MSD, Pfizer, AstraZeneca, Bristol-Myers Squibb, Daiichi Sankyo, Eli Lilly, Novonordisk, Regeneron, Roche, Sanofi

Dr. Ferrieres reports to have received grants and fees from Amgen, AstraZeneca, Merck and Sanofi.

Dr. Kastelein declares that he has acted as a consultant to and received honoraria from the following companies: Amgen, AstraZeneca, Boehringer Ingelheim, Catabasis, Cerenis, CSL Behring, Dezima Pharmaceuticals, Eli Lilly, Esperion, Isis, Merck, Novartis, Pronova, Regeneron, Sanofi, The Medicines Company, Kowa, Gemfire, Cymabay and Roche

Dr. Drexel reports to have received lecture honoraria from Amgen, AstraZeneca, Bayer, Boehringer-Ingelheim, MSD, Sanofi-aventis, and Takeda. Advisory Committee and Consultant: Amgen, Boehringer-Ingelheim, Genericon, MSD, Novartis, Takeda

Martin Horack reports to have no conflict of interest.

Dr. Bramlage reports to have received consultancy fees and / or research funding from Bayer, Daiichi Sankyo, Edwards Lifesciences, Harvest Technologies, Hexal, Merck, Pfizer, Novartis, Sanofi, Servier, Takeda, and UCB. He also received funding for the drafting the paper.

Dr. Lautsch, Dr. Brudi, Dr. Vanneste, Dr. Chazelle, Dr. Sazonov, Dr. Ambegaonkar are employees of Merck & Co., Inc., Kenilworth, NJ.

We confirm that the manuscript has been read and approved by all named authors and that there are no other persons who satisfied the criteria for authorship but are not listed. We further confirm that the order of authors listed in the manuscript has been approved by all of us.

We confirm that we have given due consideration to the protection of intellectual property associated with this work and that there are no impediments to publication, including the timing of publication, with respect to intellectual property. In so doing we confirm that we have followed the regulations of our institutions concerning intellectual property.

We further confirm that any aspect of the work covered in this manuscript that has involved either experimental animals or human patients has been conducted with the ethical approval of all relevant bodies and that such approvals are acknowledged within the manuscript.

We understand that the Corresponding Author is the sole contact for the Editorial process (including Editorial Manager and direct communications with the office). He/she is responsible for communicating with the other authors about progress, submissions of revisions and final approval of proofs. We confirm that we have provided a current, correct email address which is accessible by the Corresponding Author and which has been configured to accept email from the journal.

Signed by all authors as follows:

Peter Bramlage Date: 15.09.2016 Signature: ____________________

on behalf of the coauthors. Signatures will be provided to the journal as soon as possible.
